# The Study of the Prevention of Anal Cancer (SPANC): design and methods of a three-year prospective cohort study

**DOI:** 10.1186/1471-2458-13-946

**Published:** 2013-10-09

**Authors:** Dorothy A Machalek, Andrew E Grulich, Richard J Hillman, Fengyi Jin, David J Templeton, Sepehr N Tabrizi, Suzanne M Garland, Garrett Prestage, Kirsten McCaffery, Kirsten Howard, Winnie Tong, Christopher K Fairley, Jennifer Roberts, Annabelle Farnsworth, I Mary Poynten

**Affiliations:** 1 The Kirby Institute, The University of New South Wales, Sydney 2052, NSW, Australia; 2 The Western Sydney Sexual Health Centre, University of Sydney, Westmead Hospital, Westmead 2145, NSW, Australia; 3 RPA Sexual Health, Royal Prince Alfred Hospital, Camperdown 2050, NSW, Australia; 4 Regional HPV Labnet Reference Laboratory, Department of Microbiology and Infectious Diseases, The Royal Women's Hospital, Parkville 3052, Victoria, Australia; 5 Murdoch Children's Research Institute, Parkville 3052, Victoria, Australia; 6 Department of Obstetrics and Gynaecology, University of Melbourne, Parkville 3052, Victoria, Australia; 7 School of Public Health, University of Sydney, Sydney 2006, NSW, Australia; 8 Centre for Applied Medical Research, St Vincent's Hospital, Sydney 2010, NSW, Australia; 9 Melbourne Sexual Health Centre and the School of Population Health, University of Melbourne, 580 Swanston Street, Carlton 3053, Victoria, Australia; 10 Douglass Hanly Moir Pathology, 14 Giffnock Avenue, Macquarie Park 2113, NSW, Australia

**Keywords:** Human papillomavirus, Anal cancer, Methods, Natural history, Men who have sex with men, Homosexual, Anal squamous cell carcinoma, HSIL/HGAIN, Precancerous conditions, Cancer screening

## Abstract

**Background:**

The incidence of human papillomavirus (HPV)-associated anal cancer is increasing in men who have sex with men (MSM). Screening for the presumed cancer precursor, high-grade anal squamous intraepithelial lesions (HSIL) in a manner analogous to cervical cancer screening has been proposed. Uncertainty remains regarding anal HPV natural history and the role of anal cytology and high-resolution anoscopy (HRA) as screening tests. Well-designed cohort studies are required to address these issues.

**Methods/design:**

The SPANC study is a prospective study of the epidemiology of low-risk and high-risk anal HPV infection and related cytological and histological abnormalities in HIV-negative and HIV-positive homosexual men aged 35 years and over. The study aims to recruit 600 men from community-based settings in Sydney, Australia. There are six study visits over three years. At the first five visits men undergo a digital ano-rectal examination (DARE), an anal “Papanicolaou” (Pap) test for HPV detection, genotyping and anal cytology, followed by HRA and directed biopsy of any visible abnormalities. The men also complete a behavioural questionnaire before each visit. Questions include a detailed history of sexual behaviour, of anal symptoms, possible anal cancer risk factors and validated quality of life and psychosocial questions. Questionnaires are also completed 2 weeks and 3 months following the provision of test results and include questions on participant experience during the procedure and post-procedure symptoms, including pain and bleeding in addition to quality of life/ psychosocial outcomes.

**Discussion:**

Recruitment for the study began in September 2010 and will conclude in mid-2015, with follow up continuing to 2018. Thus far, over 350 men have been recruited from a variety of community-based settings and are broadly representative of the target screening population. The SPANC study is one of only a small number of cohort studies globally to perform HPV, cytology and HRA screening on all participants over multiple time points. The study results will contribute to understanding of the natural history of anal HPV and inform the possible development of guidelines for implementing anal cancer screening programs in this population.

## Background

More than 80% of squamous cell anal cancers are caused by infection with high-risk human papillomavirus (Hr-HPV), mainly HPV16 [1]. In the general population anal cancer is uncommon, with rates of between 1 and 2 cases per 100,000 per year in most settings [2]. However, certain subpopulations are at increased risk of this disease. These include; 1) women who have had previous HPV-associated anogenital (i.e. vulval, vaginal or cervical) HSIL or cancer [3]; 2) people with immune deficiency, including those with HIV infection and organ transplant recipients [4]; and 3) men who have sex with men (MSM) [5]. Anal cancer rates are highest in MSM [6], especially in HIV-positive MSM, with no evidence of decline in incidence since the introduction of effective antiretroviral therapy [7].

Prophylactic vaccination against HPV has enormous potential to prevent anal cancer among high-risk populations in the future [8,9]. However, current adult populations are unlikely to benefit from this. In view of the increasing health burden of anal cancer and its biological similarities to cervical cancer, some researchers have advocated for the introduction of an anal cancer screening program for high-risk populations [10]. There is uncertainty though, regarding anal HPV natural history, especially concerning rates of progression and regression of the presumed anal cancer precursor, high-grade squamous intraepithelial lesions (HSIL). There is ongoing assessment and discussion around the performance of anal cytology. In most countries, there is a scarcity of specialist clinicians trained in high resolution anoscopy (HRA). Furthermore, the safety and efficacy of available treatment options is yet to be proven in randomised controlled trials [11]. For these reasons, despite years of advocacy, no national authorities recommend a cytology-based screening program for anal cancer in any high-risk population [12].

Little is known about the psychosocial and quality of life (QoL) impacts of anal cancer screening. The psychological and QoL burden of anal cancer cytology screening among homosexual men is especially important since a large number of men will have abnormalities detected at screening [13]. In women, there is strong evidence that psychological wellbeing, QoL and psychosexual health are negatively affected by the experience of an abnormal cervical screening and/or HPV test result [14]. It is currently unknown how people will respond to an anal cancer screening program and research examining the psychosocial consequences of screening is needed.

The Study of the Prevention of Anal Cancer (SPANC) is a longitudinal study exploring the epidemiology of anal HPV infection and related abnormalities among a community recruited cohort of homosexual men. The study results will contribute to our understanding of the natural history of the disease, the psychological well-being and QoL impact and the health-related costs of anal cancer screening of homosexual men. This paper describes the main design and methodological aspects of the study and includes descriptive data on selected baseline characteristics for the first 350 participants recruited from September 2010 to the end of July 2013.

## Design and methods

### Overview of design

SPANC is an ongoing, prospective, cohort study based in Sydney, Australia. The study follows HIV-negative and HIV-positive homosexual men aged 35 years and older at six visits over a period of three years, including a baseline enrollment visit, four follow-up screening visits and final visit to discuss all study results. The main objective of the study is to investigate the epidemiology of anal HPV infection and related cytological and histological anal abnormalities in this population. Specifically, the study aims to 1) determine the prevalence, incidence and risk factors for anal HPV genotype detection and associated lesions, 2) investigate rates of clearance and persistence of anal HPV infection, 3) investigate rates of progression and regression of intraepithelial anal lesions and 4) assess the psychosocial/QoL impact of screening in homosexual men. In addition to the objectives described above, a number of sub-studies are nested within the main study. These include sub-studies on molecular biomarkers, lesion-specific HPV genotyping using laser capture microdissection (LCM), HPV serology, immunology, health economics and qualitative studies. Details of these sub-studies will not be covered in this paper.

The baseline data collection commenced in September 2010 and will conclude when the full study sample of 600 men is enrolled (approximately July 2015). Data from the baseline phase of the study will be used to investigate the prevalence of type-specific anal HPV infection and prevalence of anal squamous intraepithelial lesions. Results from the baseline questionnaire will be used to investigate the demographic, behavioural and clinical determinants and risk factors for type-specific HPV infection and low and high-grade anal squamous intraepithelial lesions (LSIL and HSIL). Additionally, the baseline cytology and HPV genotyping results will be compared with the final histological diagnosis to evaluate the value of anal cytology and HPV genotyping as potential screening tools in a setting where the prevalence of anal HPV and HSIL is very high [13]. Baseline psychosocial factors will be compared with post-test questionnaire findings.

Follow-up of participants will continue to 2018. Incidence, persistence, clearance and regression rates of type-specific HPV infection and associated HSIL will be calculated and compared between groups with different behavioural, biological and clinical risk factors. Additionally, the impact of screening on men’s physical and psychological well-being and QoL will be measured and used to assess the benefits and potential harms of the screening test.

The study will recruit a total of 600 men with an approximate 3:2 ratio of HIV-negative to HIV-positive participants. Interim baseline data will be published on the first 350 participants recruited to the end of July 2013.

### Ethics

Ethics approval for the study was first granted on 21 April 2010 by the St Vincent’s Hospital (SVH, Sydney, Australia) Human Research Ethics Committee (File Number 09/203). The study is conducted in accordance with the National Health and Medical Research Council (NHMRC) National Statement of Ethical Conduct in Human Research 2007 (http://www.nhmrc.gov.au/book/national-statement-ethical-conduct-human-research), the World Medical Association Declaration of Helsinki (October 2000) and the International Conference on Harmonisation Note for Guidance on Good Clinical Practice (ICH-GCP) (http://www.tga.gov.au/industry/clinical-trials-note-ich13595.htm). Written informed consent is obtained from all individuals before any study-specific procedures are performed.

### Study population and recruitment

Obtaining a representative sample of homosexual men is challenging, as there is no defined population from which to draw a sample. Therefore, recruitment strategies used in SPANC are based on previous community-based studies conducted by the Kirby Institute and are centered on a number of community-based settings in Sydney. These include gay community social events and organizations, referrals from other participants and participants from previous community-based studies involving homosexual men at the Kirby Institute. Information regarding the study is distributed through leaflets and advertisements on gay community websites and SPANC information nights. To ensure a diverse population of men is reached, advertisements are also placed in the local gay press and the social networking site Facebook.

Eligible participants are men aged 35 years or over who report sex with another man in their lifetime (any homosexual contact). Participants are excluded if they are unable to understand English, unable to attend scheduled follow-up visits or are unwilling to undergo HRA, have a bleeding disorder or are on anti-coagulation medications (except aspirin and other non-steroidal anti-inflammatory drugs). Men who have previously had an HRA or who have a history of anal cancer are also excluded from the study.

Once enrolled, contact with the participants is maintained though regular reminder emails, text messages and telephone calls from SPANC study staff. To maximize retention and maintain engagement with the cohort, participants receive regular SPANC newsletters containing information on the progress of the study as well as general information relevant to the field. The newsletters are also used to address issues and concerns raised by the participants at their study visits or in the feedback section of the follow-up questionnaires.

### Study procedures

**Figure 1:**
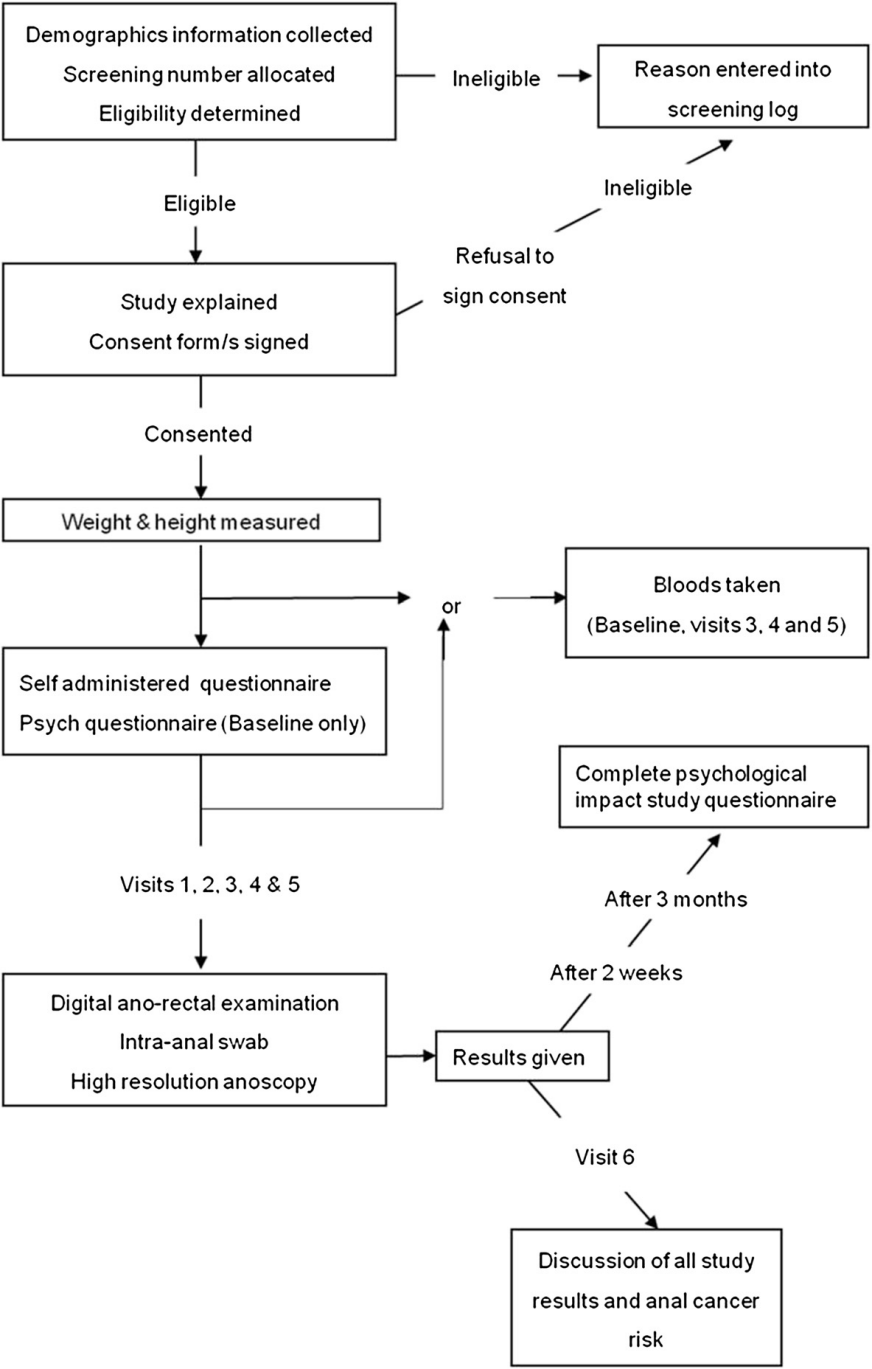
Study flow at each study visit.

At each screening visit men undergo a digital ano-rectal exam (DARE), an anal Pap test for HPV genotyping and anal cytology, HRA and biopsy of any visible abnormalities. Testing for sexually transmitted infections is performed on blood, urine, throat and rectal samples at all visits other than the six month visit. All participants complete audio, computer-assisted, self-administered interviews (ACASI, QDS, Bethesda, MD) at each visit. Additionally, two home-based self-administered questionnaires are scheduled two weeks and three months after the provision of cytology and histology results. The study flow and visit schedule are outlined in Figure 1 and Table 1. The final home based self-administered questionnaire takes place six months after Visit 5, to assess any longer term cancer worry and psychological distress that may be related to anal cancer screening. Two months after Visit 5, participants undergo a 30–60 minute consultation with a research clinician (visit #6). At this study visit, the results of each study visit (#1 - #5), including cytological and histological assessments and HPV testing, are summarised and explained to participants. In addition, individualised evaluation of anal cancer risk based on their test results, and information about future potential anal cancer screening options are provided.

**Table 1:** Study visit schedule outlining procedures and assessments performed at each visit

	**Baseline**	**Follow-up *(+/− 3 months)***				
**Visit number**	**1**	**2**	**3**	**4**	**5**	**6**
**Study months**	**0**	**6**	**12**	**24**	**36**	**38**
***Interview process***						
Informed consent	**X**					
Baseline interview	**X**					
Follow up Interview		**X**	**X**	**X**	**X**	
Psychological interview	**X**	**X**	**X**	**X**	**X**	
***Clinical process***						
Venepuncture	**X**		**X**	**X**	**X**	
Digital ano-rectal exam	**X**	**X**	**X**	**X**	**X**	
Anal swab	**X**	**X**	**X**	**X**	**X**	
Urine sample & throat swab^1^	**X**		¶	¶	¶	
High resolution anoscopy & biopsy	**X**	**X**	**X**	**X**	**X**	
Discussion of all study results and anal cancer risk						**x**
***Laboratory process***						
HIV testing^2^	**X**		¶	¶	¶	
Syphilis testing ^1^	**X**		¶	¶	¶	
HPV PCR	**X**	**X**	**X**	**X**	**X**	
Anal cytology	**X**	**X**	**X**	**X**	**X**	
Histology^3^	**X**	**X**	**X**	**X**	**X**	
Gonorrhoea and chlamydia testing^1^	**X**		¶	¶	¶	

^1^Syphilis, anal, pharyngeal and urethral gonorrhoea, and anal and urethral chlamydia testing: participants who reported sexual activity since the last annual visit undergo anal STI testing at the follow up visit (as indicated by ¶). ^2^HIV testing: only HIV-negative participants. Follow up testing is performed in HIV-negative men who report sexual activity since the last HIV test (as indicated by ¶). ^3^Biopsy and histology: participants with lesions seen at high resolution anoscopy undergo biopsy for further histological assessment.

### Questionnaires

Data collected in the baseline questionnaire (visit #1) includes data on socio-demographics, predictors of gay social engagement and community involvement, lifetime and recent (previous six months) sexual practices, general health, lifestyle and anal health. At follow-up visits (#2 - #5) the questions focus on sexual experiences, general health, lifestyle and anal health issues in the previous six months. Additional information is sought from HIV-positive participants at all study visits on clinical measures such as CD4 T-lymphocyte count, HIV viral load and AIDS-defining illnesses. Measures of the potential psychosocial impact of anal screening are collected at the baseline visit and in the two-week and three-month (post-visit) questionnaires. The two-week questionnaire also contains questions on discomfort experienced during the procedure and post-procedure symptoms, including pain and bleeding.

An overview of the components of the questionnaire, including examples of variables and the measures and scales utilized are provided in Table 2.

**Table 2:** Summary of the main sections of the baseline and follow up questionnaires

**Section**	**Examples of variables**	**Measures and standardized scales**
**Socio-demographics**	Age, country of birth, ethnicity, relationship status, accommodation status, employment status, income	Adapted from the Health in Men [15] and Positive Health [16] studies
**Sexual practices**	Sexual identity	Adapted from Health in Men and Positive Health studies [15,16]
	Age at first receptive anal intercourse:	Gay community periodic surveys [17,18]
	Number of lifetime insertive and receptive anal partners (with and without a condom)	
	Lifetime number of female partners	
	Preferred position during anal sex (receptive or insertive)	
	Number of insertive and receptive anal partners (with and without a condom) in the previous six months	
	Frequency of fingering, fisting and insertion of toys and other objects in the previous six months	
**Gay social engagement**	Time spent with gay/homosexual friends	Gay community periodic surveys [19]
	Disclosure of sexuality to friends/family	
	Number of gay friends	
	Involvement with gay community	
**Health and lifestyle**	Smoking status	45 and Up study [20]
	Alcohol and drug use	
	History of health problems (lifetime and in the last month)	
	History of depression and anxiety	
	Physical activity	
	General health	
**Section**	**Examples of Variables**	**Measures and Standardized Scales**
**Anal health**	History and frequency of anal douching	Adapted from the Blokes with Anal Squamous Intraepithelial Lesions (BASIL) study [21]
	History of STIs	
	Anal symptoms	
	HPV knowledge	
**Psychosocial evaluation**	Generic measure of health related quality of life	SF36 v2 (Australian) [22]
	Disease specific distress associated with diagnosis of SIL	Cervical Dysplasia Distress and Screening questionnaires (modified) [23,24]
	Generic measures of distress	Distress thermometer [24]
	Worry about developing anal cancer Perception of risk of anal cancer	Cancer worry [24]
	Frequency of thinking about the tests	Perceived risk of cancer Intrusive thoughts [25]
	Psychosexual health	
**HIV-positive status specific**	Length of HIV infection	Positive Health study [16]
	Viral load	
	Most recent CD4-Tcell count	
	Nadir CD4-Tcell count	
	Type of antiretroviral medication	
	Length of time on antiretroviral medication	
	History of AIDS associated illness	
**Procedure related feedback**	Post procedure pain and bleeding	
	Length of pain and bleeding	
	Discomfort associated with the procedures	
**Other**	Reason for joining study	Adapted from Health In Men and positive Health studies [15,16]
	Source of recruitment	

### Clinic procedures

#### Venepuncture

Blood samples are drawn for HIV and syphilis testing. Participants provide additional, optional consent to have their blood stored (as serum and cell pellets). HPV genotype specific serology may be performed and specimens may be tested for other potential determinants of anal cancer risk at a later stage.

#### Urine and throat swab

Swabs from the tonsillar arch and posterior nasopharynx are collected for gonorrhoea testing. For urethral gonorrhoea and chlamydia testing participants are asked to void the first 10 ml of urine into the sterile container provided.

#### Digital Ano-Rectal Examination (DARE)

DARE is performed on all participants at each study visit by a study physician using lubricant mixed with 1% lignocaine. Possible abnormalities and their positions in the anal canal or perineum are recorded on the HRA report form (See Additional file 1).

#### Anal pap test collection

Two anal canal samples are collected from all participants at each study visit. The first is an anal Pap test, wherein a Dacron swab moistened with tap water is inserted (3-5 cm) into the anal canal by the study clinician, pressed firmly against the wall of the anal canal and slowly withdrawn in a spiral fashion over approximately one minute. Immediately after sampling, the swab is inserted into a vial containing 20 ml of PreservCyt (Hologic, Inc., Marlborough, MA, United States) fixative medium and mixed vigorously. Specimens are sent to Douglas Hanly Moir Pathology (DHM, Sydney, Australia) for processing. Before cytological processing, one fifth (4 mL) of the PreservCyt sample is transferred into a second vial for HPV DNA detection and genotyping to avoid cross-contamination [26]. The remaining sample is used for cytological analysis. If the anal Pap test is unsatisfactory for cytological evaluation, a repeat anal collection is performed no less than two weeks from the first. This minimum time requirement is to allow sufficient time for the anal mucosal cells to regenerate after the initial Pap test.

A second swab (BD ProbeTec sample collection kit, BD Diagnostics, Sparks, MD, United States) is inserted (3-5 cm) into the anal canal by the study clinician and gently rotated, then placed into the sterile transport tube for testing for gonorrhoea and chlamydia.

#### High Resolution Anoscopy (HRA)

HRA is performed at each study visit following collection of the anal canal samples. After a visual examination of the perianal region and perineum, a plastic anoscope coated with lignocaine-containing lubricant is inserted into the anal canal and the introducer removed. A gauze swab soaked in 3% acetic acid is then inserted through the anoscope and left in the anal canal for one minute, to stain the anal mucosa. The gauze is then removed and the anoscope re-inserted. The anal canal is visualised under high magnification using a colposcope with real-time video image capturing software (Second Opinion Version 7.0, Second Opinion Telemedicine Solutions, Inc. Torrance, California, USA). Further acetic acid is repeatedly applied, followed by Lugol’s iodine to stain for any macroscopic abnormality [27,28]. Biopsies of visual abnormalities suspicious of HPV-related lesions are taken with mini Tischler forceps and placed into a formalin/saline solution and sent to DHM for processing and histopathological assessment. Separate forceps are used for each lesion, in order to prevent cross contamination.

Following removal of the anoscope, 3% acetic acid is applied to the perianal skin and the area examined using the colposcope. If both intra-anal and peri-anal biopsies are to be taken, the anoscopist may schedule the participant to return for peri-anal biopsies at a later visit (within two weeks of study visit). All peri-anal abnormalities are biopsied under lignocaine local anaesthesia.

Clinical impressions of intra-anal and peri-anal abnormalities and their anatomical positions are recorded on the HRA report form (See Additional file 1). Quantification of the total area of intra-anal and/or peri-anal tissue affected by HSIL is recorded retrospectively once the histology results are known using a seven point scale; 1) Single small lesion, 2) < half of one quadrant, 3) ≥ half of one quadrant but < one quadrant, 4) ≥ one quadrant but < two quadrants, 5) ≥ two quadrants but < three quadrants, 6) ≥ three quadrants, 7) circumferential disease. Additionally, annotations of suspected HSIL lesions are taken at the time of the procedure. All reporting is performed with the knowledge of the participant’s HIV status. The clinicians are also aware of the participant’s cytology, and HRA +/− biopsy results from any previous study visits.

All study anoscopists are required to undergo a formal training program prior to their involvement with the study. This training program was adapted from the British Society for Colposcopy and Cervical Pathology Training Program [27]. Anoscopists are required to observe at least 25 HRA procedures and then perform at least 50 HRAs under the direct supervision of an experienced anoscopist. All cases are recorded in a log book and the results of anal Pap tests and biopsies reviewed, when they become available. Once the anoscopist is deemed competent, they start performing HRA on their own. If required, they are able to access advice and clinical support from an experienced anoscopist on an ongoing basis and the log book of all procedures is maintained.

A quality assurance program for anoscopists is also in place. First, each anoscopist’s cytology/histology correlation and number of unsatisfactory Pap tests are summarised quarterly and a meeting of anoscopists is held at which the data are discussed and any significant differences between anoscopists are addressed. Second, a SPANC multidisciplinary team meeting is held quarterly involving the study investigators, anoscopists and pathologists. Six to eight cases are covered at each meeting including review of HRA images, cytology and histology slides and results, and HPV results. Each anoscopist presents their cases and any discordant results are discussed by the team. Detailed minutes are kept and action plans determined.

### Laboratory procedures

#### HPV genotyping

All anal swabs placed in PreservCyt specimens are tested for HPV DNA using the Roche LINEAR ARRAY (LA) HPV genotyping test (Roche Molecular Systems, Alameda, CA, United States). DNA is extracted with the automated MagNA Pure isolation and purification system (Roche Molecular Systems) by a modified protocol using 1 ml aliquots of PreservCyt specimens as described previously [29]. The LA HPV genotyping test involves PCR amplification of a 450 bp region of the HPV L1 gene and allows for the identification of 37 HPV genotypes (6, 11, 16, 18, 26, 31, 33, 35, 39, 40, 42, 45, 51, 52, 53, 54, 55, 56, 58, 59, 61, 62, 64, 66, 67, 68, 69, 70, 71, 72, 73, 81, 82, 83, 84, IS39 (HPV 82v subtype), and CP6108 (HPV 89) as well as amplification of 265 bp region of the human ß-globin gene, serving as an internal control. As an in-house modification, samples that produced a negative internal control are retested with ½ the volume of eluted DNA in order to reduce the inhibition due to high bacterial DNA content in those samples. In addition, due to possible cross-reactivity of the HPV-52 probe with types 33, 35, and 58 amplicons, samples positive for one or more of HPV-33, 35, and 58 probes are further tested using a real-time PCR assay with an HPV-52 specific hydrolysis probe to rule out presence of HPV 52 in addition to those types [30].

#### Syphilis testing

A screening enzyme immunoassay (EIA; ICE syphilis, Murex Biotech Ltd, Dartford, United Kingdom) is used to test for syphilis. Positive screening results are confirmed with the Treponema pallidum particle agglutination assay and/or the fluorescent treponemal antibody absorption test. The rapid plasma reagin (RPR) test is used on those with confirmed syphilis to assist clinical staging and to detect re-infection. All participants are tested for syphilis at the baseline visit. At visits 3, 4 and 5, serology is performed on those participants who have previously tested negative for syphilis and report sexual activity since the last visit. In participants with confirmed syphilis at any prior visit, only RPR is performed.

#### HIV testing

HIV testing is performed at baseline on all participants who report their HIV status to be negative, using the AxSYM HIV antigen/antibody Combo (Abbott Diagnostics, Abbott Park, IL, United States). Positive results are confirmed by a western blot assay. HIV testing is performed at visits 3, 4 and 5, on those HIV-negative participants who report sexual activity since the last visit.

#### Gonorrhoea and chlamydia testing

All baseline samples are tested for gonorrhoea and chlamydia by strand displacement amplification (SDA) using the BD ProbeTec assay (BD Diagnostics, Sparks MD). Positive samples undergo a supplementary nucleic acid amplification assay to confirm the diagnosis. Follow up testing is performed at visit 3, 4, and 5 on those participants who report sexual activity since the last visit.

### Cytology and histopathology

The laboratory reporting cytological and histopathological results is a specialist gynaecological unit operating within a large private general pathology laboratory in Sydney, Australia. The gynaecological unit reports more than 250 000 cervical cytology specimens (conventional and liquid-based slides) per year and more than 12 000 gynaecological surgical specimens per year. Three anatomical pathologists, each with more than 15 years’ experience in gynaecological pathology, are involved in the study. The number of cytologists and pathologists involved were limited in order to enhance reproducibility of reporting. Prior to commencement of the study, a pathologist and a cytologist prepared a teaching manual based on available literature and assembled a set of teaching slides, drawn from previous smaller studies of anal canal lesions. All participating staff undertook training based on these materials.

#### Cytopathology

Prior to processing for cytology, a 4 ml aliquot is removed from the ThinPrep vial (total sample volume 20 ml) under sterile conditions. This aliquot is forwarded to Royal Women’s Hospital Molecular Microbiology laboratory (Melbourne, Australia) for HPV testing. The remainder of the sample is processed using a ThinPrep 2000 processor (Hologic Inc.,Marlborough, MA, United States) to produce a TP slide (Hologic Inc., Marlborough, MA, United States), which is then stained with the ThinPrep proprietary stain (Hologic Inc., Marlborough, MA, United States). The slide is manually screened by a study cytologist on a standard light microscope. The cytologist completes a checklist, and creates a provisional report, which is forwarded with the slide to one of the three study pathologists for assessment and final reporting. A ‘satisfactory’ slide is defined as having at least 2000 nucleated squamous cells. The presence or absence of a transformation zone component (at least 10 colorectal glandular cells and/or squamous metaplastic cells) is recorded but does not affect determination of satisfactory status. The Bethesda System (TBS) [31] criteria and terms are used for cytology reporting. Both the cytologist and pathologist view the slide blind to any knowledge of previous or current cytology or histology. Participant date of birth and study code was available, but no other demographic or clinical information accompanied the specimen.

#### Histopathology

Biopsies are received in 10% formal saline and processed in a routine fashion. Nine levels of each biopsy are prepared. Levels one to three and seven to nine are routinely stained with Haematoxylin and Eosin (H&E) stain. Levels four to six are prepared on a coated slide and left unstained for potential use for histochemical staining or immunostaining. Each case is viewed and reported by one of the three study pathologists. Every attempt is made to ensure that a different pathologist views the concurrent cytology and histology specimens of an individual participant. At times where this is not possible due to logistical issues, the study pathologist reports the cytology and histology at different times. All reporting is done blinded to clinical factors (other than age) and previous results.

Reporting of the biopsies is performed in accordance with criteria, terminology and recommendations of the Lower Anogenital Squamous Terminology (LAST) Project [32,33]. Details relevant to this study are as follows:

• With respect to non-invasive HPV-related disease, the following terms are used: Negative for Squamous Intraepithelial lesion (SIL); Low-grade SIL (LSIL) and High-grade SIL (HSIL)

– Within the category of HSIL, further subdivision is performed, into anal intraepithelial neoplasia (AIN) grade 2 and AIN grade 3. If the biopsy is from perianal skin the terms perianal intraepithelial neoplasia (PAIN) 2 and 3 are used.

• Immunostaining for p16 INK4A (p16) is performed on the unstained spare slide in the following circumstances:

– To differentiate HSIL from a benign mimic, in particular, atypical immature metaplasia, but also inflammatory/reactive changes, tangential sectioning and partial epithelial denudation.

– When a diagnosis of AIN 2 is proposed, in order to confirm positive p16 reactivity. If the result is negative, the lesion is downgraded to LSIL or negative for SIL.

• Immunostaining for p16 is not used in formulating diagnoses of straightforward LSIL or straightforward HSIL – AIN 3.

• Immunostaining for p16 is reported as positive if there is “continuous strong nuclear or nuclear plus cytoplasmic staining of the basal cell layer with extension upwards involving at least one third of the epithelial thickness” [32,33]. Any other pattern of staining is regarded as negative.

• Carcinomas are measured in two dimensions and categorized as “Superficially invasive squamous cell carcinoma of the anus” (SISSCA) if they meet the LAST definition.

In addition to the LAST categories above, a decision was made to sub-categorise LSIL into ‘exophytic’ and ‘flat’, based on presence or absence respectively of architectural features of condylomata accuminatum. This decision reflected the possibility that the two variations of LSIL may be associated with different HPV types [34,35].

If infection with organisms other than HPV is suspected, appropriate stains are used, for instance immunoperoxidase staining for HSV-1 and/or HSV-2 in the presence of ulceration; and Ziehl-Neelsen staining if granulomata are present.

### Characteristics of the enrolled population

The socio-demographic and lifestyle characteristics of the first 350 participants are presented in Table 3. Data are presented for the overall cohort, and stratified by HIV status. Details of HIV infection (if positive) are presented in Table 4. Since the study’s commencement approximately 594 men were contacted by the study team at least once and approximately 404 were screened for eligibility. Fifty-four (54) men were excluded because they did not meet the inclusion criteria. Most common reasons for exclusion were: too young (26%); unable to attend scheduled visits due to time/location (26%); have previously had a HRA (13%); have a history of bleeding disorders or were on anti-coagulant medication (11%); have previously been diagnosed with anal cancer (9%), or other reason (15%). By the end of July 2013, 350 men of whom 29% (n=101) were HIV-positive were recruited into the study.

**Table 3:** Socio-demographic, health and lifestyle characteristics

	**Overall (n=350)**		**HIV positive (n=101)**		**HIV negative (n=249)**	
	n	%	n	%	n	%
**Age**						
Median (IQR)	49 (42–55)		49 (43–54)		49 (42–56)	
Mean (SD)	49.6 (9.3)		48.7 (7.4)		49.9 (9.9)	
35 – 44 years	110	31.4	31	30.7	79	31.7
45 – 54 years	143	40.9	47	46.5	96	38.6
55 + years	97	27.7	23	22.8	74	29.7
**Country of birth**						
Australia	234	66.9	72	71.3	162	65.1
New Zealand	21	6.0	6	5.9	15	6.0
Other	95	27.1	23	22.8	72	28.9
**Ethnic background**						
Anglo- Celtic	292	83.4	80	79.2	212	85.1
Aboriginal/Torres Strait	5	1.4	2	2.0	3	1.2
Other	53	15.1	19	18.8	34	13.7
**Highest level of education**						
High School	67	19.1	24	23.8	43	17.3
Tertiary diploma or trade^1^	84	24.0	28	27.7	56	22.5
Undergraduate degree	86	24.6	28	27.7	58	23.3
Postgraduate degree	113	32.3	21	20.8	92	37.0
**Work situation**						
Full/Part time/self-employed	254	72.6	66	65.4	188	75.5
Unemployed	17	4.9	4	4.0	13	5.2
Retired	57	16.3	22	21.8	35	14.1
Other ^2^	22	6.3	9	8.9	13	5.2
**Weekly income (AU$)**						
<200	24	6.9	7	6.9	17	6.8
200-499	59	16.9	29	28.7	30	12.1
500-999	61	17.4	23	22.8	38	15.3
1000—1499	61	17.4	12	11.9	49	19.7
≥1500	140	40.0	29	28.7	111	44.6
*Missing responses*	*5*	*1.4*	*1*	*1.0*	*4*	*1.6*
**Currently in a relationship**						
No	155	44.3	54	53.5	101	40.6
Yes, with male partner	191	54.6	45	44.6	146	58.6
Yes, with female partner	4	1.1	2	2.0	2	0.80
**Smoking status**						
Never	193	55.1	44	43.6	149	59.8
Past	108	30.9	35	34.7	73	29.3
Current	49	14.0	22	21.8	27	10.8
**Alcohol consumption**						
Never	28	8.0	9	8.9	19	7.6
< once a week	81	23.1	33	32.7	48	19.3
1-2 days a week	86	24.6	17	16.8	69	27.7
3-4 days a week	61	17.4	21	20.8	40	16.1
5-6 days a week	51	14.6	12	11.9	39	15.7
Every day	43	12.3	9	8.9	34	13.7
**Recreational drug use in the past 6 months **^3^						
No	95	27.1	19	18.8	76	30.5
Yes	255	72.9	82	81.2	173	69.5

^1^Technical and further education; ^2^Other includes: Student, looking after family/friend, disabled/sick, doing unpaid work; ^3^Drug use includes; marijuana, amyl, cocaine, ecstasy, speed, crystal methamphetamine, LSD, Special K, heroin, GHB, Viagra, other drugs.

**Table 4:** Characteristics of HIV-positive men

	**n**	**%**
**Years HIV-positive**		
Median (Range)	16 (8.3-25.3)	
Mean (SD)	16.5 (9.0)	
**Nadir CD4 T cell/μL**		
Over 500	11	10.9
201 - 500	41	40.6
51 - 200	27	26.7
< 50	21	20.8
Missing	1	1.0
**Last CD4 T cell/μL**		
Over 750	29	28.7
501-750	36	35.6
351-500	20	19.8
201-350	9	8.9
101-200	1	1.0
50-100	1	1.0
<50	2	2.0
Missing	3	3.0
**Last viral load test**		
Undetectable	80	79.2
Detectable	14	13.9
Missing	7	6.9
**History of AIDS defining illness**		
No	70	69.3
Yes	31	30.7
**Currently taking any anti-retroviral medication**		
No	11	10.9
Yes	90	89.1

The majority of men were recruited from community based sources including from gay community events (32%) or the SPANC website/Facebook (13%). Fifteen percent (15%) were recruited through friends or partners, 14% through gay organizations or HIV/AIDS organisations, 11% through gay press and 6% other. In 9% of cases men were recruited from general practice or sexual health clinics.

The median age of participants at the time of baseline interview was 49 years (IQR 42–55). The majority of participants (67%) were born in Australia and most (83%) reported Anglo-Celtic ethnic background. The cohort was highly educated, with 57% of participants having completed university and 40% reporting a weekly income of ≥ $1500. Forty-five percent (45%) of men reported being a past or current cigarette smoker. The majority of men (92%) consumed alcohol at least once a week and 73% have used drugs recreationally in the past 6 months (Table 3).

HIV-negative participants in SPANC were generally more likely to have higher levels of education, higher income, having full or part time employment and higher alcohol consumption. HIV-positive participants were more likely to report past or current cigarette smoking and recreational drug use (Table 3).

Almost all participants self-identified as being homosexual, gay or queer (95.4%) and 4.6% self identified as bisexual, heterosexual or other. The median age of first receptive anal intercourse was 21 years (IQR 17–25). Participants were highly sexually active, with 53% of the men reporting more than 200 lifetime male sexual partners, and 29% reported having more than 10 male sexual partners in the previous 6 months. Fifty five percent (55%) of men reported having unprotected receptive anal intercourse (URAI) in the previous 6 months. A lifetime history of anal STIs was also common in this group being reported by 26%, 21%, 17% and 19% of men for anal gonorrhea, syphilis, chlamydia and herpes, respectively. Having ever had anal or genital warts was reported by 43% and 29% of men, respectively. Compared with the HIV-negative, HIV-positive participants in SPANC reported more lifetime and recent sexual partners, more frequent URAI, and more past STIs.

Among HIV-positive participants (n=101), the median time they had been living with HIV was 16 years (IQR 8–25). The majority (84%) had a self-reported CD4 T cell count over 350cells/μL, 79% reported undetectable HIV viral load at their last test and 89% were on treatment at the time of the baseline interview (Table 4).

## Discussion

SPANC is a prospective longitudinal study of the epidemiology of anal HPV and related abnormalities in a community-based cohort of homosexual men aged 35 and older in urban Sydney, Australia. Recruitment for the study began in September 2010 and will conclude in mid-2015, with follow-up continuing to 2018. By July 2013, approximately 404 men had been assessed for inclusion and over 350 men have been recruited into the study. SPANC participants are recruited from a variety of community-based settings and can be considered as broadly representative of the potential target screening population. Men older than 35 years are targeted because anal cancer is still rare at younger ages [2] and at present, older men are unlikely to benefit from the implementation of HPV vaccination. Thus any future community-based screening program would most likely focus on older homosexual men.

Ultimately, the goal of anal cancer screening will be to identify those pre-cancerous lesions most likely to progress to cancer. One of the key issues in HSIL natural history studies is defining what “high-grade” lesions mean clinically, and which high-grade lesions are at highest risk of persistence. It is reasonable to assume that those lesions that persist over 3 years of follow-up are those that are at highest risk of progression to cancer. As most HSIL will never progress to anal cancer [13], identifying risk profiles and correlates of persistence among disease categories is the first step in establishing a better surrogate endpoint for anal cancer risk. This is crucial to ensure that only those at highest risk of progressing to anal cancer receive treatment.

### Strengths and limitations of the study

The strength of the SPANC study is that it is one of only a small number of cohort studies globally to perform both cytology and HRA screening on all participants over multiple time-points, as well as performing HPV DNA genotyping. As both anal cytology and HRA are known to under-diagnose HSIL [36], performing both is critical for the most accurate research-based estimate of the extent of disease. Also, extremely detailed sexual behaviour and other health and lifestyle behaviour data are collected, which will allow excellent characterisation of associations of these factors with anal HPV and related lesions.

Currently, there are no widely accepted guidelines on how anal cancer prevention programs should be implemented. Anal cancer screening only occurs in selected specialised clinics in a small number of countries, not including Australia. As a result, the efficient conduct of SPANC has been hindered by significant infrastructure constraints. Recruitment into the study has been slower than expected, as clinical and laboratory infrastructure has had to be developed by the research team. A shortage of medical personnel trained to perform HRA and the lack of designated clinic space and facilities for these procedures have been major issues.

The SPANC study is a multidisciplinary collaboration, bringing together researchers skilled in epidemiology, behavioural science, nursing, laboratory virology, immunology, sexual health, cytopathology, histopathology, psychology, and health economics. The study results will provide evidence to inform the possible development of guidelines for implementing screening programs in this population. Additionally, the SPANC study is a unique opportunity to address the lack of resources and infrastructure in Australia and to establish an advocacy agenda for additional anal cancer/HSIL diagnostic services for those populations with recognised higher risks of anal cancer.

## Competing interests

AEG has received honoraria and research funding from CSL Biotherapies, honoraria and travel funding from Merck, and sits on the Australian advisory board for the Gardasil HPV vaccine. CKF has received honoraria, travel funding and research funding from CSL and Merck, sits on the Australian advisory board for the Gardasil HPV vaccine, and owns shares in CSL Biotherapies. SMG have received advisory board fees and grant support from CSL and GlaxoSmithKline, and lecture fees from Merck, GlaxoSmithKline and Sanofi Pasteur; in addition, has received funding through her institution to conduct HPV vaccine studies for MSD and GlaxoSmithKline and is a member of the Merck Global Advisory Board as well as the Merck Scientific Advisory Committee for HPV. RJH has received support from CSL Biotherapies and MSD. All other authors declare that they have no competing interests.

## Authors’ contributions

DAM (PhD student) drafted the manuscript and performed the descriptive data analysis. AEG (Principal Investigator) conceived of the study, participated in its design, analysis and interpretation of data and reviewed the manuscript for important intellectual content. RJH (Principal Clinical Investigator) participated in study design, helped draft the manuscript and reviewed the manuscript for important intellectual content. FJ (Co-Investigator and Data Manager) participated in study design, analysis and interpretation of data and reviewed the manuscript for important intellectual content. DJT (Co-Investigator and Study Clinician) participated in the study design, helped draft the manuscript and reviewed the manuscript for important intellectual content. SNT (Co-Investigator) participated in the study design, helped draft the manuscript and reviewed the manuscript for important intellectual content. SMG, GP, KMc, KH, CKF, AF (Co-Investigators) participated in the study design and reviewed the manuscript for important intellectual content. WT (Study Clinician) reviewed the manuscript for important intellectual content. JR (Co-Investigator) participated in the study design, helped draft the manuscript and reviewed the manuscript for important intellectual content. IMP (Co-Investigator and Project Leader) participated in the study design and coordination and helped draft the manuscript. All authors read and approved the final manuscript.

## Pre-publication history

The pre-publication history for this paper can be accessed here:

http://www.biomedcentral.com/1471-2458/13/946/prepub

## Supplementary Material

Additional file 1 SPANC HRA report form. Click here for file
