# Supplementary material for: The Study of the Prevention of Anal Cancer (SPANC): design and methods of a three-year prospective cohort study
Source: BMC Public Health. 2013 Oct 9;13:946. doi: 10.1186/1471-2458-13-946 (PMC3852594; doi:10.1186/1471-2458-13-946)
Supplement: Additional file 1 — SPANC HRA report form. [file 1471-2458-13-946-S1.pdf]

The Study of the Prevention of Anal Cancer (SPANC): design and methods of a three-year prospective cohort study

Additional Material

High resolution anoscopy report form

Study of the Prevention of Anal Cancer (SPAN)

A study of the natural history of anal human papillomavirus
Infection and anal cellular abnormalities in homosexual men

Time In:

Time Out:

High Resolution Anoscopy report form

Study ID: ____________ **Name code***(first name-last name):* ☐☐--☐☐ **Date of HRA performed** *(DD/MM/YYYY):* ______/_______/_______

Digital Rectal Examination Impression:
Prostate:

- ☐ **Normal**
- ☐ **Smooth enlargement**
- ☐ **Irregular/rocky enlargement**
- ☐ **Tender**

Other Abnormalities: ☐ R ☐ RP ☐ RA ☐ P ☐ A ☐ LP ☐ LA ☐ L

A - Mass
B – Indentation/ulcer
C – Other, please state.....................................

HRA Intra-anal: ☐ **Entirely Normal**

☐ R ☐ RP ☐ RA ☐ P ☐ A ☐ LP ☐ LA ☐ L

Site Specific Abnormalities:

	Acetwhite /Cobblestng/Puctn/Prmnt Vssls/Lugol's neg/Other							Biopsies A	Biopsies B	Biopsies None	Clinical Impression
Right Lateral	☐	☐	☐	☐	☐	☐	1	☐	☐	☐	☐
Right Anterior	☐	☐	☐	☐	☐	☐	2	☐	☐	☐	☐
Anterior	☐	☐	☐	☐	☐	☐	3	☐	☐	☐	☐
Left Anterior	☐	☐	☐	☐	☐	☐	4	☐	☐	☐	☐
Left Lateral	☐	☐	☐	☐	☐	☐	5	☐	☐	☐	☐
Left Posterior	☐	☐	☐	☐	☐	☐	6	☐	☐	☐	☐
Posterior	☐	☐	☐	☐	☐	☐	7	☐	☐	☐	☐
Right Posterior	☐	☐	☐	☐	☐	☐	8	☐	☐	☐	☐

Clinical Impression key: 1 Normal, 2 Wart, 3 LSIL, 4 HSIL, 5 other, specify above
